# Supplementary material for: Multi-Omic Profiles in Infants at Risk for Food Reactions
Source: Genes (Basel). 2022 Nov 3;13(11):2024. doi: 10.3390/genes13112024 (PMC9690066; doi:10.3390/genes13112024)
Supplement: Supplementary file 1 [file genes-13-02024-s001.zip › genes-2002420-supplementary.pdf]

Supplemental Table S1. Saliva Molecular Profiles.

	V	P-value	Adj. P-value
Cytokine			
IL-18	1893.5	0.30231	0.69709
CXCL10	1480	0.35742	0.69709
IL-18/IL-6	1854	0.39834	0.69709
IL-8	1804	0.54318	0.71671
IL-6	1673.5	0.99622	0.99622
microRNA			
hsa-miR-203b-3p	2913	0.004377	0.043774
hsa-miR-30e-3p	2478	0.27789	0.67357
hsa-miR-148a-3p	2450	0.3313	0.67357
hsa-let-7d-3p	1977	0.34563	0.67357
hsa-miR-375-3p	1986	0.36462	0.67357
hsa-miR-26a-5p	2007	0.41141	0.67357
hsa-miR-16-5p	2037	0.4841	0.67357
hsa-miR-21-5p	2058	0.53886	0.67357
hsa-miR-146b-5p	2323	0.64814	0.72016
hsa-miR-27b-3p	2160	0.84086	0.84086
Gene			
FLG	2639.5	0.051623	0.25811
STAT6	2503	0.14271	0.3224
SERPINB2	2435	0.19344	0.3224
SPINK5	2349.5	0.49984	0.62479
MALT1	2234	0.91162	0.91162
Bacteria Phyla			
Cyanobacteria	1515	0.004845	0.048452
Elusimicrobia	1714	0.044438	0.22219
Fusobacteria	1855	0.15044	0.30752
Actinobacteria	2552	0.16598	0.30752
Proteobacteria	1886	0.18944	0.30752
Bacteroidetes	1895	0.20205	0.30752
Firmicutes	2516	0.21526	0.30752
Candidatus Saccharibacteria	2463	0.30573	0.38216
Spirochaetes	1988	0.36893	0.40992
Tenericutes	2294	0.73483	0.73483
Viral Phage			
Proteus virus PM135	2955	0.002528	0.027808
Streptococcus phage K13	2707	0.044011	0.22184
Erwinia phage vB_EamM_Kwa	1780	0.081472	0.22184
Shewanella sp. phage 1/4	2633	0.086562	0.22184
Mycobacterium virus Cooper	2615	0.10084	0.22184
Streptococcus phage T12	2563	0.15275	0.28005
Aeromonas virus 65	1940	0.27431	0.38955
Streptococcus phage phiARI0	1945	0.28331	0.38955
Streptococcus phage EJ-1	1968	0.32728	0.40001
Bacillus virus Moonbeam	2344	0.58815	0.64696
Escherichia virus phiX174	2100	0.65692	0.65692
